# Exercise Counterbalances Rho/ROCK2 Signaling Impairment in the Skeletal Muscle and Ameliorates Insulin Sensitivity in Obese Mice

**DOI:** 10.3389/fimmu.2021.702025

**Published:** 2021-06-21

**Authors:** Vitor R. Muñoz, Rafael C. Gaspar, Matheus B. Severino, Ana P. A. Macêdo, Fernando M. Simabuco, Eduardo R. Ropelle, Dennys E. Cintra, Adelino S. R. da Silva, Young-Bum Kim, José Rodrigo Pauli

**Affiliations:** ^1^ Laboratory of Molecular Biology of Exercise, University of Campinas (UNICAMP), Limeira, Brazil; ^2^ Multidisciplinary Laboratory of Food and Health, School of Applied Sciences, State University of Campinas, Limeira, Brazil; ^3^ Laboratory of Nutritional Genomics, University of Campinas (UNICAMP), Limeira, Brazil; ^4^ Postgraduate Program in Rehabilitation and Functional Performance, Ribeirão Preto Medical School, University of São Paulo (USP), Ribeirão Preto, Brazil; ^5^ School of Physical Education and Sport of Ribeirão Preto, University of São Paulo (USP), Ribeirão Preto, Brazil; ^6^ Division of Endocrinology, Diabetes and Metabolism, Beth Israel Deaconess Medical Center and Harvard Medical School, Boston, MA, United States

**Keywords:** obesity, insulin sensitivity, exercise, Rho-kinase (ROCK), skeletal muscle

## Abstract

Physical exercise is considered a fundamental strategy in improving insulin sensitivity and glucose uptake in skeletal muscle. However, the molecular mechanisms underlying this regulation, primarily on skeletal muscle glucose uptake, are not fully understood. Recent evidence has shown that Rho-kinase (ROCK) isoforms play a pivotal role in regulating skeletal muscle glucose uptake and systemic glucose homeostasis. The current study evaluated the effect of physical exercise on ROCK2 signaling in skeletal muscle of insulin-resistant obese animals. Physiological (ITT) and molecular analysis (immunoblotting, and RT-qPCR) were performed. The contents of RhoA and ROCK2 protein were decreased in skeletal muscle of obese mice compared to control mice but were restored to normal levels in response to physical exercise. The exercised animals also showed higher phosphorylation of insulin receptor substrate 1 (IRS1 Serine 632/635) and protein kinase B (Akt) in the skeletal muscle. However, phosphatase and tensin homolog (PTEN) and protein-tyrosine phosphatase-1B (PTP-1B), both inhibitory regulators for insulin action, were increased in obesity but decreased after exercise. The impact of ROCK2 action on muscle insulin signaling is further underscored by the fact that impaired IRS1 and Akt phosphorylation caused by palmitate in C2C12 myotubes was entirely restored by ROCK2 overexpression. These results suggest that the exercise-induced upregulation of RhoA-ROCK2 signaling in skeletal muscle is associated with increased systemic insulin sensitivity in obese mice and further implicate that muscle ROCK2 could be a potential target for treating obesity-linked metabolic disorders.

## Introduction

The metabolic condition of insulin resistance precedes type 2 diabetes mellitus and has generated a high number of deaths worldwide (1, 2). In this scenario, considering that the mechanisms related to insulin resistance are not fully understood, it is relevant to investigate new potential regulators of insulin-stimulated glucose uptake in peripheral tissues (e.g., skeletal muscle) (3–6).

The Rho-kinase (ROCK) protein is relevant in several physiological processes, such as gene expressions, inflammation, cytoskeleton organization, and smooth muscle contraction (7). The mechanism of activation and inhibition involves the binding of GTPase enzymes to the binding domain of ROCK, with RhoaAGPase activating and RhoE GTPase inhibiting (8, 9). Previously, our work revealed that, in response to insulin stimulation, ROCK was activated by RhoA GTPase, leading to IRS1 phosphorylation in serine 632/635 and increasing the insulin signal pathway and glucose uptake in skeletal muscle (10). In addition, the deficiency of ROCK1 impaired the skeletal muscle insulin signaling and declined the systemic insulin sensitivity (11). Altogether, these findings suggested that ROCK1 exerts fundamental action on insulin signaling in skeletal muscle and collaborates notoriously for glycemic homeostasis of the organism.

Exercise can prevent and treat type 2 diabetes arising from obesity by improving insulin sensitivity through increased glucose uptake in skeletal muscle (12–14). We recently showed that swimming exercise increases ROCK1 and ROCK2 contents and ROCK activity, improving insulin sensitivity and GLUT4 translocation in the gastrocnemius muscle of lean rats (15). Otherwise, the ROCK inhibition impaired these positive effects after physical exercise in lean rats (15). In addition, we observed that middle-aged Fischer rats, an aging model without significant adipose mass gain, demonstrated lower ROCK2 content and ROCK activity but without ROCK1 modulation in the skeletal muscle compared to young rats (16). Thus, it remains unclear whether increased glucose uptake and insulin sensitivity induced by exercise are linked to ROCK2 signaling in skeletal muscle. This study investigated whether the effects of aerobic training on insulin sensitivity and glycemia in obese animals were associated with the ROCK signaling pathway.

## Materials and Methods

### Animal Characterization

Four-week-old Swiss mice (weighing 15–20 g) were provided by the Central Animal Laboratory from Unicamp (CEMIB). All animal experiments were approved by the Ethics Committee on Animal Use (CEUA) of the University of Campinas, under protocol number 2805-1.

### Maintenance of Animals

The animals were kept in cages under controlled conditions of luminosity, with a cycle of 12 hours of exposure to light and twelve hours in the absence of light. The animals had free access to water and food (standard or high-fat diet). The standard diet consisted of fat from soybean oil (5 g), protein (23 g), and carbohydrates (51.5 g/100 g of diet – 339 Kcal). The high-fat diet (HFD, PragSoluções^®^, 58,35% of fat) was composed of 35.8 g of fat from coconut oil, 23 g of protein, and 34.5 g of carbohydrates (totalizing 552.2 Kcal), as described previously (17, 18).

### Incremental Load Test to Determine the Exhaustion Velocity

The mice were previously adapted to the running ergometer. During adaptation, rodents remained on the treadmill for 10 minutes at a speed of 3 m/min. This adaptation was carried out for five consecutive days. Then, the incremental load test was performed. During the evaluation, there was an increase in the workload every 3 min, with a 3m/min speed increase. It was considering that the animals reached exhaustion when the rodents were no longer able to stay running at the established speed, and they touched the treadmill back wall more than five times with an interval of 1 minute. The maximum speed obtained in the incremental test was considered for the prescription of aerobic exercise. This protocol was adapted from a previous study (19).

### Experimental Groups and Exercise Protocol (Aerobic Training)

**Figure 1:**
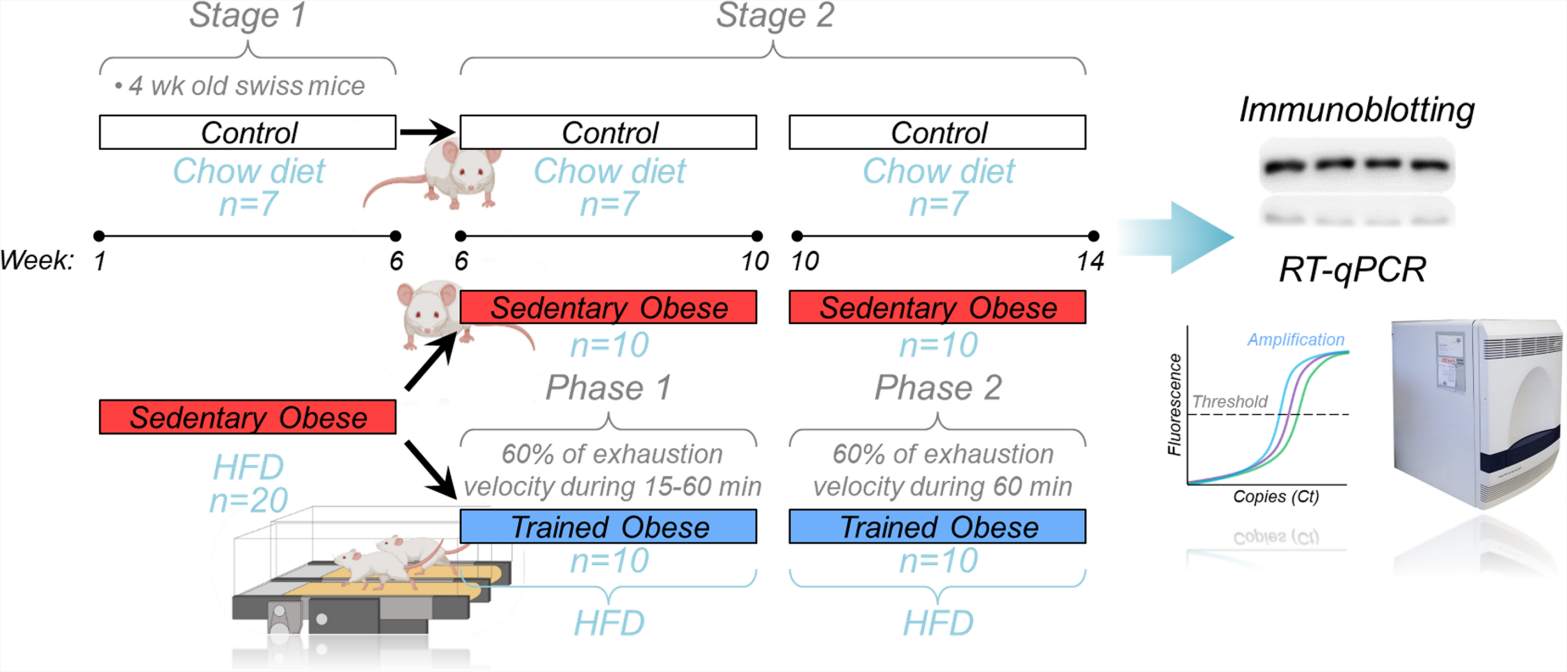
Experimental design: Schematic design showing the whole experiment divided into two stages. In Stage 1, the obese animals (red bar) were fed an HFD for 6 weeks. After that (Stage 2), the sedentary obese group was divided into two groups (sedentary and trained), where trained animals performed aerobic training on the treadmill. During Phase 1 of the aerobic training, the exercise session duration was increased weekly (15-60 minutes) at 60% of the exhaustion velocity. Phase 2 was performed at 60% of the exhaustion velocity for 60 minutes/day. After the 14 weeks of the experiment, the skeletal muscle samples were collected to perform the molecular analysis (immunoblotting and RT-qPCR).

Mice were distributed in the following groups (7 rodents/group): control (C), sedentary obese (OB), and trained obese (TOB). The aerobic training was composed of eight weeks, which was distributed into two phases: Phase 1 (6-10^th^ week), lasted four weeks, and in this period, the volume was increased gradually (15 min per week up to 60 min per week); Phase 2 (10-14^th^ week), which the volume of last week of the physical training (from Phase 1) was maintained for the next four weeks (until the end of study). The animals exercised for five consecutive days, followed by two days of rest. The physical training started after the induction of obesity, which was performed over six weeks with the mice being fed with an HFD. Before starting the training protocol, the fasting glycemia was analyzed, and an insulin tolerance test was performed. Therefore, the entire experiment period lasted for 14 weeks (**Figure 1**).

### Insulin Tolerance Test (ITT)

The insulin tolerance test (ITT) was performed 24 hours after the last physical training session. All animals fasted for 8 hours before the test. For this purpose, the mice received an intraperitoneal injection of human recombinant insulin (Humulin R) from Eli Lilly (Indianapolis, IN, USA) at a concentration of 0.75 U/kg of body weight. After a small cut at the end of their tails, the blood was collected for glucose analysis. Blood collection was performed at baseline (fasting stage and before receiving insulin) and at 30, 60, 90, and 120 minutes after receiving insulin injection. By collecting and analyzing blood glucose at different test times, it was possible to obtain the area under the glucose curve (20).

### Euthanasia of Animals

Before tissue extractions, mice received an injection of chlorohydrate ketamine (50 mg/kg Ketalar; Parke-Davis, Ann Arbor, MI) and xylazine (20 mg/kg; Rompun, Bayer, Leverkusen). Anesthetics were administered to the animals’ peritoneum. All animals fasted for 8 hours before the tissue extraction procedures.

### Tissue Extraction, Homogenization, and Determination of Total Protein Content

Skeletal muscle samples (gastrocnemius) were taken 10 min after insulin injection (intraperitoneally injection: 10 U/kg of body weight). The control/negative animals received saline. Therefore, 10 min after insulin/saline injection, muscle tissue samples were harvested and later homogenized in extraction buffer at 4°C with a Polytron (Brinkmann Instruments model PT 10/35) at maximum speed for 30 s. After incubating for 1 h, the extracts were centrifuged at 11,000 rpm, with the temperature maintained at 4°C (Palo Alto, CA) for 15 min. Part of the sample was used to determine the total protein content using bicinchoninic acid (BCA), and another part was used to perform the immunoblotting (IB) technique using specific antibodies. The epididymal, mesenteric, subcutaneous, and retroperitoneal adipose tissues were removed and weighed on an analytical balance to compare groups.

### Immunoblotting – IB

After determining the total protein content, the Laemmli was added to the supernatant buffer (21) containing 100 mM DTT and heated in boiling water for 5–10 minutes. Next, equal amounts of protein from the gastrocnemius (50 µg) were used for application in polyacrylamide gel and separation by electrophoresis. The membranes were incubated with: antibodies against: phospho-Y972 IR (#GTX25678) from GeneTex^®^; phospho-Y612 IRS1 (#44816G) from Life Technologies^®^; Akt (#4685), phospho-Ser473 Akt (#9271), phospho-Ser9 GSK3β (#5558), GSK3β (#5676), phospho-Ser632/635 IRS1 (#2388), RhoA (#2117), PDK (#3062), phospho-Ser241 PDK (#3061), PTEN (#9188), phospho-Ser380 PTEN (#9151), and GAPDH (#2118) from Cell Signaling Techonology^®^; phospho-T696 MYPT1 (#92590) from Millipore^®^; ROCK2 (#5561), PTP1B (#14021) from Santa Cruz Biotechnology^®^; RhoE form Sigma^®^. Antibodies were diluted 1:1000. After overnight incubation with primary antibody, the membranes were washed with TBS+Tween 0.05% for 30 min, incubated with horseradish peroxidase secondary antibodies (1:2000 dilution; Cell Signaling Techonology^®^) for 1 h and washed again with TBS+Tween 0.05% for 30 min. The bands were visualized with enhanced chemiluminescence and quantified by an Image J program (v1.52a, NIH).

### ROCK Activity Assay

The Rho-kinase activity was evaluated by measuring its substrate phosphorylation (pMYPT1) using 100 µg of muscle lysate, 1 mM of ATP, and 500 ng of recombinant MYPT1 (Millipore^®^). This mixture was incubated for 30 min under agitation at 30°C. The reaction was stopped using 2x Laemmli and submitted to IP protocol. The membrane was incubated using anti-pMYPT1 (T696) from Millipore^®^.

### Quantitative Real-Time PCR

The RNA was obtained from skeletal muscle (gastrocnemius) with TRIzol (ThermoFischer^®^, MA, USA) and submitted to cDNA conversion (High Capacity cDNA Reverse Transcription Kit; ThermoFischer^®^, MA, USA). The quantitative Real-Time PCR was performed using the 7500 Fast Real-Time machine (Applied Biosystems^®^, CA, USA) with SYBR Green PCR Mastermix (Applied Biosystems^®^, CA, USA). The mRNA quantification for each gene was normalized with *Gapdh* using the 2^−ΔΔCT^ method. The primer sequences are listed in **Table 1**.

**Table 1:** Primer sequence list.

*S/c2a4-R Mouse*	Reverse	CGGTCAGGCGCTTTAGACTC
*S/c2a4-F Mouse*	Forward	ATCATCCGGAACCTGGAGG
*Hk2-R Mouse*	Reverse	GGAAGCGGACATCACAATC
*Hk2-F Mouse*	Forward	AGAGAACAAGGGCGAGGAG
*Gpi1-R Mouse*	Reverse	CCCGATTCTCGGTGTAGTTG
*Gpi1-F Mouse*	Forward	ATGGGCATATTCTGGTGGAC
*Pfkm-R Mouse*	Reverse	TTCCTGTCAAAGGGAGTTGG
*Pfkm-F Mouse*	Forward	CTGGTGCTGAGGAATGAGAA
*Pfkp-R Mouse*	Reverse	TCCCACCCACTTGCAGAAT
*Pfkp-F Mouse*	Forward	AAGCTATCGGTGTCCTGACC
*Tpi1-R Mouse*	Reverse	CGGTGGGAGCAGTTACTAAA
*Tpi1-F Mouse*	Forward	TATGGAGGTTCTGTGACTGGA
*Pgk1-R Mouse*	Reverse	CTTTAGCGCCTCCCAAGATA
*Pgk1-F Mouse*	Forward	GAGCCTCACTGTCCAAACTA
*Pgam1-R Mouse*	Reverse	GTACCTGCGATCCTTGCTGA
*Pgam1-F Mouse*	Forward	GACGATCTTATGATGTCCCACC
*Eno1-R Mouse*	Reverse	GAAGAGACCTTTTGCGGTGT
*Eno1-F Mouse*	Forward	CTTGCTTTGCAGCGATCCTA
*Eno3-R Mouse*	Reverse	CTTCCCATACTTGGCCTTGA
*Eno3-F Mouse*	Forward	CTGTGCCTGCCTTTAATGTG
*Pkm-R Mouse*	Reverse	CAACAGGACGGTAGAGAATGG
*Pkm-F Mouse*	Forward	CTGTGGAGATGCTGAAGGAG
*Pcx-R Mouse*	Reverse	GCAATCGAAGGCTGCGTACAGT
*Pcx-F Mouse*	Forward	GGATGACCTCACAGCCAAGCAT
*ldh3a-R Mouse*	Reverse	GCCATGTCCTTGCCTGCAATGT
/*dh3a-F Mouse*	Forward	GCAGGACTGATTGGAGGTCTTG
*Ogdh-R Mouse*	Reverse	ATCCAGCCAGTGCTTGATGTGC
*Ogdh-F Mouse*	Forward	GGTGTCGTCAATCAGCCTGAGT
*Gapdh-R Mouse*	Reverse	ACACATTGGGGGTAGGAACA
*Gapdh-F Mouse*	Forward	AACTTTGGCATTGTGGAAGG

### Cell Culture

C2C12 myoblasts (ATCC^®^ CRL-1772™) were grown in Dulbecco’s Modified Eagle’s Medium (DMEM - ThemoFisher scientific^®^ #12100046) containing 10% of fetal bovine serum (FBS - Gibco^®^ #A476680) 1% of Penicillin/Streptomycin complexes (P/S – Gibco^®^ #15140122) at 37°C, 5% CO2 up to ~ 80% of confluence. Then, the differentiation process was induced by replacing the FBS growth medium with 2% horse serum (HS - Gibco^®^ #26050070), and the culture medium was replaced every two days. The analyses were performed on day 6 of differentiation.

### Insulin Resistance Assay in C2C12 Myotubes

On the 5th day of differentiation, C2C12 myotubes were treated with Bovine Serum Albumin Free Fat Acid (BSA FFA Sigma Aldrich^®^ #A8806-5G) to compound control group, or 500 µM of Palmitate conjugated to BSA FFA (Sigma Aldrich^®^ #P0500) for 24 h to promote insulin resistance. After this treatment, on the 6th day of differentiation, these cells were treated with 200 nM of Insulin (Sigma Aldrich^®^ #I3536) for 20 minutes.

### ROCK Activity Inhibition and ROCK2 Transfection in C2C12 Myotubes

A pharmacological ROCK inhibitor (Y-27632, Cayman Chemical^®^ #129830-38-2) was used to inhibit ROCK activity at a concentration of 15 µM for 4h, during treatment with BSA FFA - Palmitate. For the overexpression of ROCK2, a GFP-mROCK2 plasmid was obtained from Addgene^®^ (#101296), where mammalian ROCK2 was cloned into a pEGFP-C1 vector. According to the manufacturer’s instructions, the transfection was performed on the 4th day of differentiation with the Lipo Plus reagent (Thermofisher Scientific #15338100). After 24 hours, cells were treated with palmitate (500 µM for 24 h) and stimulated with insulin (200 nM for 20 minutes).

### Statistical Analysis

The graphs were expressed as mean ± SEM. Data normality was analyzed using the Shapiro-Wilk test. The two groups’ comparison was analyzed using the Student *t*-test (normal) or Mann-Whitney test (non-normal). Analysis of variance - ANOVA test (normal) followed by Tukey’s or Kruskal-Wallis (non-normal) was adopted when three groups were compared. The statistical significance was set at p < 0.05. The software GraphPad Prism version 6.0 was used to perform the analysis.

## Results

### Physiological Parameters

**Figure 2:**
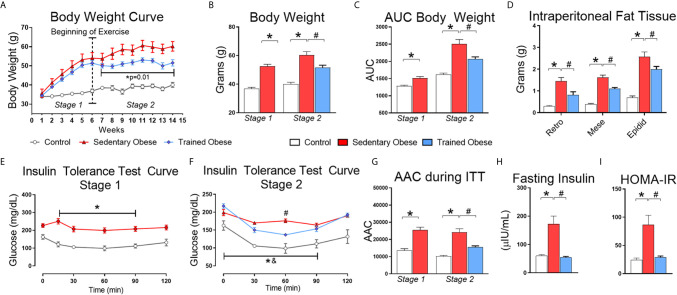
Physiological Parameters: **(A)** Body weight curve throughout the experiment, which was distributed into two stages: the first stage (1–6 weeks) performed only to induce obesity and insulin resistance; the second stage (6–14 weeks) for treatment. **(B)** Final Body weight of stages 1 and 2. **(C)** The area under the body weight curve. **(D)** Intraperitoneal from retroperitoneal, mesenteric, and epididymal fat. **(E)** The insulin tolerance test curve after the first stage (6 weeks fed a high-fat diet), performed only to confirm the insulin resistance induction. **(F)** Insulin tolerance test curve after the second stage (8 weeks fed with HFD and treatment with physical exercise for eight more weeks). **(G)** Area above the curve during the Insulin tolerance test in the first and second stages. During the first stage, 7 animals were used in the control group and 20 animals for the obese group. In the second stage, the obese group was distributed into sedentary and trained groups, where each group consisted of 10 animals. **(H)** Values of fasting insulinemia. **(I)** HOMA-IR index values. The Student t-test was used to compare two means and one-way ANOVA to compare 3 means obtained during the second stage of the experiment. All bars represent mean and SEM of Control, Sedentary Obese, and Trained Obese Mice. *p < 0.05 between Control and Sedentary Obese group. ^#^p < 0.05 between Sedentary Obese and Trained Obese groups. ^&^p < 0.05 between Trained Obese and Control group.

The animals from the obese group gained more body weight and intraperitoneal fat than the control group (**Figures 2A–D**). After six weeks of HFD feeding, we confirmed that mice fed an HFD were insulin-resistant compared to the control mice (**Figures 2E, I**). Following the obesity and insulin resistance induction period, a set of obese mice began the physical exercise for eight weeks. Exercised obese mice reduced weight gain and intraperitoneal fat compared to sedentary obese mice (**Figures 2A–D**). As expected, physical exercise improved insulin sensitivity in obese mice (**Figures 2E–I**). Serum insulin levels were increased in obese mice and restored to normal levels by exercise (**Figure 2H**).

### ROCK2, Insulin Signaling and Glucose Metabolism Genes in Gastrocnemius Muscle of Trained Obese Mice

**Figure 3:**
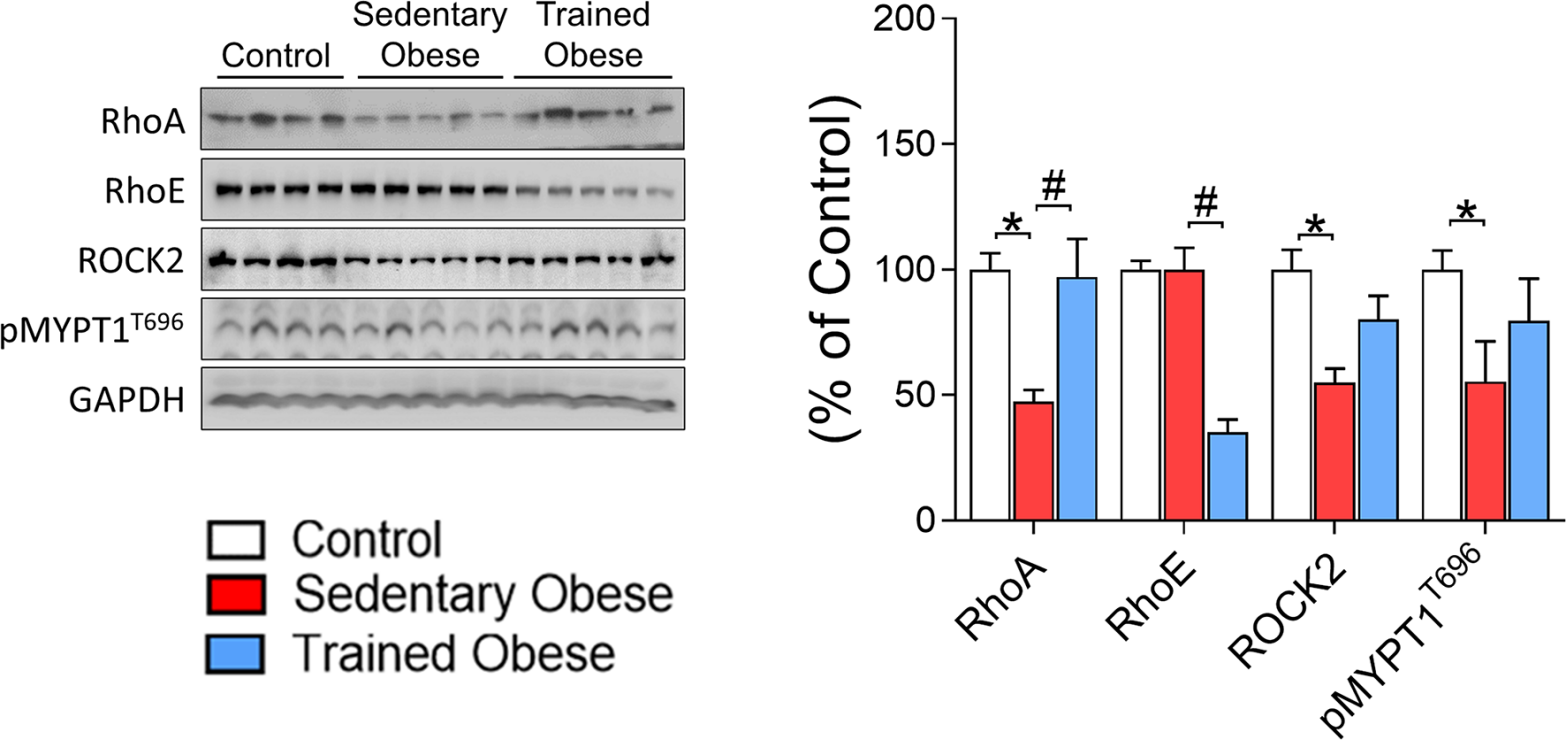
Rock signaling in the Skeletal Muscle of obese and trained obese mice: Analysis of Rock signaling (RhoA/RhoE/ROCK2/pMYPT1). The analysis was performed on percentage change related to the control group. The comparison of the means was performed using one-way ANOVA, and the p-value was set at p < 0.05. The total protein GAPDH was used as an assay control. All bars represent mean and SEM of Control, Sedentary Obese, and Trained Obese mice. *p < 0.05 between Control and Sedentary Obese group. ^#^p < 0.05 between Sedentary Obese and Trained Obese groups.

**Figure 4:**
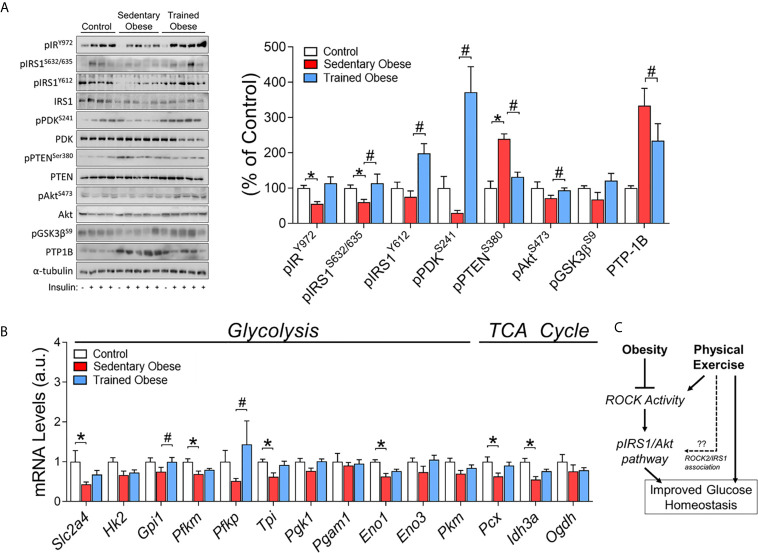
Insulin signaling in the Skeletal Muscle of obese and trained obese mice: **(A)** Phosphorylation of crucial proteins from insulin signaling pathway (pIR/pIRS1/pPDK/pPTEN/pAkt/pGsk3β/PTP-1) in skeletal muscle of control, sedentary obese and trained obese animals. The control group was composed of 1 animal that was not stimulated with insulin (-) and 3 animals that were stimulated with insulin (+); the sedentary and trained obese groups were composed of 1 animal without stimulation with insulin (-), and 4 animals stimulated with insulin (+). **(B)** Glucose metabolism-related genes (*Slc2a4, Hk2, Gpi1, Pfkm, Pfkp, Tpi, Pgk1, Pgam1, Eno1, Eno3, Pkm, Pcx, Idh3a, Ogdh*). **(C)** Schematic figure of the mechanism involving obesity, physical exercise, and Rock activity. The analysis was performed on percentage change related to the control group. The comparison of the means of stimulated groups was performed using one-way ANOVA, and the p-value was set at p < 0.05. The total proteins IRS1, PDK, PTEN, Akt, and α-tubulin were used to assay control. All bars represent mean and SEM of Control, Sedentary Obese, and Trained Obese mice. *p < 0.05 between Control and Sedentary Obese group. ^#^p < 0.05 between Sedentary Obese and Trained Obese groups.

Besides evaluating whether diet-induced obesity reduces the Rho-ROCK2 signaling pathway in skeletal muscle, we tested the possibility that physical exercise can restore this effect. We found that the amount of ROCK2 protein in skeletal muscle was significantly decreased in obese mice compared with control mice (**Figure 3**). Also, the level of RhoA protein in skeletal muscle, an upstream mediator of ROCK2, was reduced by obesity (**Figure 3**). Interestingly, these effects were completely restored to normal levels after the physical training. Along with these data, similar results were observed in MYPT1 phosphorylation as an indicator for ROCK activity and IRS1 serine 632/635 phosphorylation, which are ROCK’s phosphorylated sites (i.e., obesity decreased these parameters, but exercise increased them) (**Figures 3**, **4A**). However, obesity did not affect skeletal muscle RhoE protein, but physical exercise reduced its content (**Figure 3**).

As expected, insulin signaling components (pIR/pIRS1) stimulated by insulin were decreased in the skeletal muscle of obese mice fed an HFD. On the other hand, the exercise increased pIRS1, pPDK, and pAkt in these mice (**Figure 4A**). Obesity also led to a significant increase in PTEN phosphorylation, a negative regulator of PI3K, but exercise training suppressed this result (**Figure 4A**). Concurrently, PTP-1B protein content was decreased in trained obese mice (**Figure 4A**).

The modulations in ROCK signaling were accompanied by changes in glucose metabolism-related genes (glycolysis and TCA cycle) in the skeletal muscle. The obese mice showed lower levels in the mRNA of *Slc2a4*, Pfkm, *Tpi*, *Eno1*, *Pcx*, and *Idh3a* (**Figure 4B**). Instead, the physical exercise restored these levels and increased the mRNA levels of *Gpi1* and *Pfkp* (**Figure 4B**). Collectively, these results suggest that exercise training has beneficial effects on insulin sensitivity and glucose metabolism by increasing the expression of critical molecules associated with Rho-ROCK2 signaling, which could contribute to increased insulin-sensitizing effects in the skeletal muscle in obese mice (**Figure 4C**).

### *In Vitro* ROCK Inhibition and Overexpression in C2C12 Myotubes

**Figure 5:**
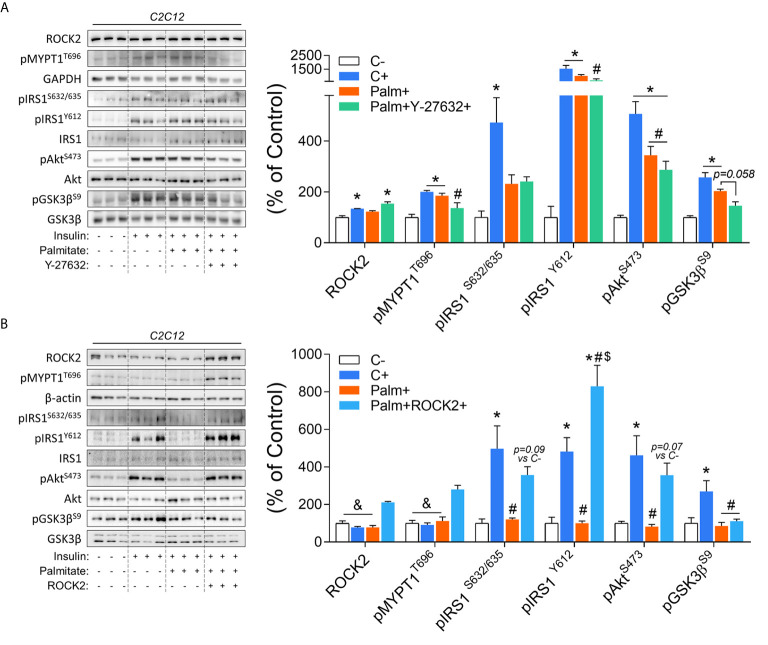
Insulin signaling in palmitate-induced insulin-resistant C2C12 myotubes in response to ROCK activity inhibition (Y-27632) and ROCK2 overexpression: **(A)** Phosphorylation of critical proteins involved with ROCK activity and insulin signaling pathway (pIRS1/pAkt/pGsk3β) in response to Insulin 200 nM for 20 minutes, Palmitate 500 µM for 24 hours, and Y-27632 15 µM for 4 hours. **(B)** Phosphorylation of critical proteins involved with Rock activity and insulin signaling pathway (pIRS1pAkt/pGSK3β) in response to Insulin 200 nM for 20 minutes, Palmitate 500 μM for 24 hours, and ROCK2 transfection. All bars represent the means and SEM of Control (C), Palmitate (Palm), Palm+Y-27632, and Palm+ROCK2 cells.*p < 0.05 *vs*. Control group without insulin (C-). ^#^p < 0.05 *vs*. Control group treated with insulin (C+). ^$^p < 0.05 *vs*. Palm group. ^&^p < 0.05 *vs*. Palm+ROCK2 group.

To confirm the role of ROCK in the control of the insulin signaling pathway in the context of insulin resistance, C2C12 myotubes were studied. Insulin increases ROCK activity (pMYPT1)and insulin signaling components phosphorylation, including pIRS1, pAkt, and pGsk3β. However, the treatment of C2C12 cells with palmitate impaired the insulin’s effect on pIRS1^S632/635^ and pAkt (**Figure 5A**). The co-treatment with ROCK inhibitor (Y-27632) and palmitate impaired the insulin-stimulated ROCK activity (pMYPT1) and pIRS1^Y612^ but preserved the attenuated phosphorylation of pIRS1^S632/635^ and pAkt compared to insulin-treated cells. A decreasing trend in the pGsk3β (*p=0.058*) compared to the group treated only with palmitate was also observed (**Figure 5A**). In contrast to the ROCK inhibition, ROCK2 overexpression in insulin-resistant C2C12 cells led to the restoration in the phosphorylation of the insulin signaling pathway proteins (**Figure 5B**). The ROCK2 overexpression increased both ROCK2 content and activity (pMYPT1) compared to the other groups. Overexpression of ROCK1 in C2C12 cells treated with palmitate restored impaired insulin-stimulated IRS1 and Akt phosphorylation (**Figure 5B**). These data suggest that ROCK2 overexpression is sufficient to promote insulin signaling in the muscle cells and protects against lipid-induced insulin resistance.

## Discussion

The present research investigated the beneficial effects of exercise on insulin signaling in insulin-resistant obese mice, with a particular interest in ROCK signaling. We found that insulin-resistant obese animals have a lower RhoA and ROCK2 protein content in skeletal muscle, contributing to impaired insulin signaling. Interestingly, exercise training restored the impairment of ROCK2 and insulin signaling in insulin-resistant obese mice. Combined with our previous studies showing that ROCK is an essential regulator of glucose metabolism (10) and ROCK activity is upregulated in gastrocnemius muscle of exercised rats (15), these findings suggest that the increase in whole-body insulin sensitivity in obese animals submitted to exercise includes the involvement of ROCK protein in skeletal muscle insulin signal regulation.

Although exercise training increases insulin sensitivity (22), its molecular mechanism remains to be elucidated. We tested the possibility that physical exercise-induced improvement of insulin sensitivity could be due to increased ROCK signaling in skeletal muscle of insulin-resistant obese mice. Here, we demonstrate that ROCK2 expression, a downstream target for RhoA, was significantly decreased in obese mice than lean control mice but was restored to a normal level by exercise training. Consistently, similar data were observed in trained obese mice for the proximal insulin signaling components, including IRS1 tyrosine phosphorylation, Akt phosphorylation, and PDK phosphorylation. Given that ROCK2 directly phosphorylates IRS1 serine residues (23), the elevation of exercise-induced ROCK2 protein may lead to an enhancement of IRS1 tyrosine phosphorylation *via* IRS1 serine phosphorylation. As a result, insulin sensitivity is increased, ultimately leading to metabolic improvement in insulin-resistant obese mice.

The previous discovery showed that animals with a global genetic deletion of ROCK developed insulin resistance and showed reduced skeletal muscle glucose uptake (11). These observations are further supported by the findings that insulin-stimulated ROCK activation in skeletal muscle is reduced in people with type 2 diabetes (24), along with an impairment of insulin-stimulated PI3K activity associated with IRS1 (25). From these observations, we propose that the elevation of skeletal muscle ROCK protein by exercise could increase insulin sensitivity in obesity by improving insulin signaling. Therefore, the modulation of muscle ROCK expression could lead to new treatment approaches for obesity and type 2 diabetes. Future studies involving specific deletion of Rock in skeletal muscle may reinforce the findings observed in the current investigation.

Previous studies have shown that in the condition of obesity and diabetes, there is an increase in the expression and activity of the PTP-1B protein, with consequent impairment in the phosphorylation of critical components of the insulin signaling pathway such as IR and IRS1 (26, 27). Interestingly, a recent study identified that RhoA could regulate PTP1B. Indeed, the increase in RhoA was associated with the inactivation of PTP-1B (28). Here, we found that PTP1B expression was increased in skeletal muscle of obese mice compared to control mice but was decreased by exercise training. Similar changes were observed in PTEN phosphorylation, a negative regulator of PI3K (29), during exercise in obese mice, suggesting that exercise suppresses dephosphorylation of the proximal insulin signaling components (i.e., IR, IRS1, and PI3K), resulting in increased insulin sensitivity. Concurrently, reciprocal changes for muscle RhoA expression were seen (i.e., RhoA decreased in obesity and rescued to a normal level by exercise). Altogether, these data demonstrate that exercise-induced amelioration of insulin sensitivity in the obesity state could be explained by exercise-mediated suppression of PTP1B and PTEN, at least in part, coupled with increased RhoA expression.

In response to physical exercise, PDK1^S241^ phosphorylation in skeletal muscle was significantly increased in obese mice. Given ROCK binds to PDK1 (30), it is conceivable that ROCK phosphorylates PDK1 serine residue at 241, leading to the increase of its activation. Upon PDK1 activation, Akt was stimulated, and thereby insulin sensitivity is enhanced. During the exercise training period, it is likely that the elevation of ROCK2 expression could be the factor for this event. However, additional investigations are necessary to determine whether ROCK directly phosphorylates PDK1 serine residues and ROCK-mediated PDK1 signaling defect may collaborate to develop insulin resistance.

Fat accumulation was positively correlated with impaired insulin sensitivity (31). In obese mice, exercise training markedly decreased body weight by 21.5% and fat deposits by 217% in retroperitoneal, 131% in mesenteric, and 82% in epididymal. We cannot rule out the possibility that exercise-induced insulin sensitivity improvement in obese mice with insulin resistance could be due to decreased adiposity secondarily. Finally, we confirm the direct role of ROCK2 activity in C2C12 myotubes where pharmacologic ROCK inhibition impaired insulin signaling pathway, as previously reported (10, 23). On the other hand, ROCK2 overexpression restored the phosphorylation of essential proteins of the insulin signaling pathway in palmitate-induced insulin-resistant cells.

## Conclusion

In conclusion, our results suggest that the increased insulin-sensitizing effect obtained through exercise training is linked to the up-regulation of RhoA and ROCK2 in skeletal muscle of insulin-resistant obese mice. Thus, the Rho-ROCK2 signaling axis could play a crucial role in regulating exercise-induced insulin sensitivity in skeletal muscle.

## Data Availability Statement

The datasets presented in this study can be found in online repositories. The names of the repository/repositories and accession number(s) can be found in the article/supplementary material.

## Ethics Statement

The animal study was reviewed and approved by Ethics Committee on Animal Use (CEUA) of the University of Campinas.

## Author Contributions

Author contributions: VM and RG performed the experiments. VM, Y-BK, and JP analyzed the data. VM prepared the figures. VM and JP drafted the manuscript. MS and FS performed cell culture experiments. AM, ER, DC, AS, FS, Y-BK, and JP edited and revised the manuscript. VM, Y-BK, and JP conceived and designed the research. All authors contributed to the article and approved the submitted version.

## Funding

This work was supported by grants from the FAPESP (15/26000-2; 2013/21491-2; 2016/18488-8), CNPq (306535/2017-3) and FAEPEX, and from the National Institutes of Health (R01DK083567 to Y-BK).

## Conflict of Interest

The authors declare that the research was conducted in the absence of any commercial or financial relationships that could be construed as a potential conflict of interest.
